# Structural Analysis of SMYD3 Lysine Methyltransferase for the Development of Competitive and Specific Enzyme Inhibitors

**DOI:** 10.3390/diseases10010004

**Published:** 2021-12-29

**Authors:** Dillon K. Jarrell, Kelly N. Hassell, Ilham Alshiraihi, Debbie C. Crans, Mark A. Brown

**Affiliations:** 1 Department of Bioengineering, University of Colorado Anschutz Medical Campus, Aurora, CO 80045, USA; 2 Department of Chemistry, Colorado State University, Fort Collins, CO 80523, USA; kelly.hassell@colostate.edu (K.N.H.); debbie.crans@colostate.edu (D.C.C.); 3 Cell and Molecular Biology Program, Colorado State University, Fort Collins, CO 80523, USA; mark.brown@colostate.edu; 4 Department of Clinical Sciences, Colorado State University, Fort Collins, CO 80523, USA; ilham.alshiraihi@colostate.edu; 5 Biology Department, University of Tabuk, Tabuk 71491, Saudi Arabia; 6 Graduate Degree Program in Ecology, Department of Ethnic Studies, Global Health and Health Disparities, Colorado School of Public Health, Colorado State University, Fort Collins, CO 80523, USA

**Keywords:** SET-domain proteins, histones, Rubisco-LSMT, small molecule enzyme inhibitors, colorectal, lung carcinoma

## Abstract

Lysine methylation is among the key posttranslational modifications to histones that contribute to epigenetic regulation. SMYD3 is a lysine methyltransferase that is essential for the proliferation of a range of tumorigenic cells. The findings that SMYD3 is significantly upregulated in most colorectal carcinomas, hepatocellular carcinomas, and breast cell carcinomas support a model in which its aberrant expression modifies established patterns of gene expression, ultimately driving unrestrained proliferation. Herein, we dissect the unique structural features of SMYD3 relative to other SET enzymes, with an emphasis on the implications for selective design of therapeutics for the clinical management of cancer. Further, we illustrate the ability of inhibitors targeting the SET domain of SMYD3 to reduce the viability of colorectal and lung carcinoma cells.

## 1. Introduction

Post-translational histone modifications, including methylation, acetylation, phosphorylation, and ubiquitination, modify chromatin structure to alter DNA accessibility and the resulting gene transcription. Histone lysine methylation is a predominant epigenetic mechanism in all eukaryotic organisms, and modulates a wide range of cellular functions, including cell differentiation and proliferation. Aberrant histone lysine methylation has been associated with a range of human diseases, including cancers.

Histone lysine methylation has been shown to be catalyzed exclusively by the Su(var)3–9 Enhancer of Zeste and Trithorax (SET) protein domain, which exists in over 100 human proteins [1,2,3,4,5]. The SET domain consists four sub-domains: the pre-SET (or N-SET), I-SET, core SET (or C-SET), and post-SET regions. The core SET domain comprises the majority of the enzyme active site. Its structure is very similar among all SET-domain containing enzymes, consisting of an anti-parallel β-barrel that surrounds an unusual knot-like structure [5,6,7,8,9]. This core SET region brings into close proximity the target protein and the methyl-donor S-adenosylmethionine (SAM) cofactor, and catalyzes the transfer of the SAM methyl group to the target lysine. In general, the N-SET domain stabilizes the core SET structure, and the post-SET domain contributes to the hydrophobic target protein binding pocket. Perhaps most interesting is the inserted I-SET subdomain, which varies considerably throughout the SET protein family, and appears to be important for substrate specificity.

A specific family of SET-domain containing proteins also contain the Myeloid-Nervy-DEAF-1 (MYND) domain. The MYND domain is a zinc-finger motif that further modulates protein–protein interactions, and separates the N-SET and I-SET subdomains. These SET and MYND Domain-containing proteins (SMYDs) have been shown to be essential for a wide range of developmental processes. Of particular interest is SMYD3, which is normally expressed predominantly during embryogenesis. SMYD3 mainly catalyzes H3K4 methylation, and its transcriptional regulation has been reported as a major component in RNA polymerase complex synthesis. SMYD3 can also trimethylate H3, H4, and H5, giving it a broad range of effects in the development of skeletal and cardiac muscle tissue, as well as in mediating estrogen-receptor gene expression [10,11,12,13]. In addition, SMYD3 methylates some non-histone targets, including VEGFR1 and MAP3K2, an integral protein in the Ras signaling pathway [14,15,16,17]. The overexpression of SMYD3 later in life has been associated with a range of cancers, including breast, lung, and colorectal carcinomas. Because of this, SMYD3 has drawn considerable interest by our laboratory and others as a pharmacologic target, especially using small molecule inhibition [18,19,20]. No SMYD3 inhibitors have advanced past clinical trials yet; however, several candidates have been validated in vitro, including BCI-121, Compound 29, and Inhibitor-4 [18,19,20].

In this study, we present and compare the tertiary structures of the subdomains in SMYD3 to the SET domains in other proteins to inform the development of specific small molecule inhibitors that could be useful in the understanding and treatment of SMYD3-positive cancers. We recently developed a panel of SMYD3 inhibitors, one of which (Inhibitor-4) effectively restricted the proliferation of breast cancer cells [19]. Here, we sought to identify both the conserved and unique structures of SMYD3, and to identify the favorable interactions between SMYD3 and Inhibitor-4. Furthermore, we sought to evaluate the efficacy of Inhibitor-4 in reducing the viability and proliferation of colorectal and lung carcinoma cells in vitro.

## 2. Methods

### 2.1. Crystallography

SMYD3 protein was purified, and X-ray crystallography data was acquired in our previous publications [21,22]. Our solved crystal structure of SMYD3 (UniProtKB—Q9H7B4 (SMYD3_HUMAN)) was aligned with the existing crystal structures of SET8, SET9, Rubisco-LSMT, Dim5, Clr4, viral SET, ZMYND10, and ETO [8,23,24,25,26,27,28,29] (PDB IDs 5HQ2, 1XQH, 1BXN, 1PEG, 1G6Z, 3KMA, 2D8Q, and 2PP4, respectively) using the protein data bank and PyMol^®^ software.

### 2.2. In Vitro SMYD3 Small Molecule Inhibitor Design and Experiments

Candidate SMYD3 inhibitors were designed in silico as previously described [19]. In that work, five lead candidates were purchased and tested in vitro. We identified Inhibitor-4 as an effective SMYD3 inhibitor capable of selectively reducing the viability and proliferation of breast cancer cells [19]. Here, we similarly applied Inhibitor-4 to lung (A549) and colorectal (DLD-1) cancer cell lines.

To assess overall cell viability, 3 × 10^4^ cells were plated in 6-well plates with various concentrations of SMYD3 Inhibitor-4 for 48 h. Then, cells were washed with PBS, trypsinized, and stained with Trypan blue to exclude died cells. Cells were counted using a TC20 automated cell counter. Viability curves were drawn using GraphPad Prism 6 software.

To assess the ability of the cells to proliferate and survive under long-term treatment with Inhibitor-4 (survival fraction), 1000 cells were plated in a 6-well plate, and treated with various concentrations of SMYD3 Inhibitor-4. Then, cells were incubated in a humidified incubator at 5% CO_2_ and 37 °C for two weeks. Colonies were fixed with 75% ethanol, and stained with 0.2% Crystal Violet dye. Colonies were counted, and survival fraction was calculated as the number of colonies relative to the untreated group. Curves were drawn using GraphPad Prism 6 software.

## 3. Results

**Figure 1:**
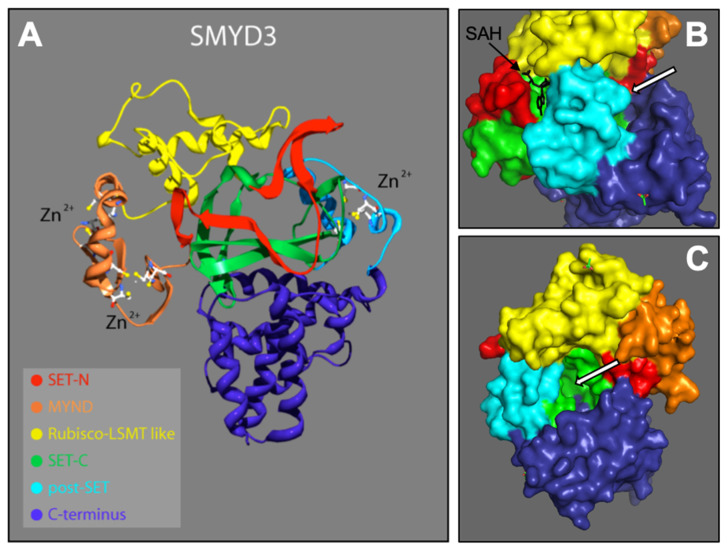
**SMYD3 structure.** 2.3 Å crystallized resolution. Colors represent regions of SMYD3 secondary structure: N-SET, C-SET, MYND, Rubisco-LSMT (I-SET), C-Terminus [16,17]. (**A**) Ribbon model, including Zinc atoms important for stability. (**B**) Surface model depicting SAH (methyl donor cofactor) in its native binding pocket. White arrros point to target protein bnding pocket. (**C**) Rotated surface model depicting target protein binding pocket.

From the N- to C-terminal, SMYD3 consists of six subdomains: the N-SET, MYND, I-SET, core SET (C-SET), post-SET, and C-terminus domains. These domains are depicted according to our 2.3 Å solution (Figure 1A). SMYD3 contains two functional binding pockets: one for the cofactor SAM/SAH (Figure 1B), and one for the protein target (Figure 1B,C). With the exception of the C-terminus, we conducted detail sequence/structure analyses for each of these subdomains, and compared the structures to similar subdomains in other SET proteins across various species. The C-terminus domain is the least conserved among the SMYD family, and shows no significant similarity to other known protein domains. It consists of a series of helices that we predict may be involved in homo- or hetero- protein interactions. As it is structurally well separated from the active site, C-terminal associations are unlikely to affect the methyltransferase activity of SMYD3, and thus, reduces its promise as a site for enzyme inhibitor targeting.

### 3.1. N-SET, C-SET, and Post-SET Domains Highly Similar among SET Enzymes

**Figure 2:**
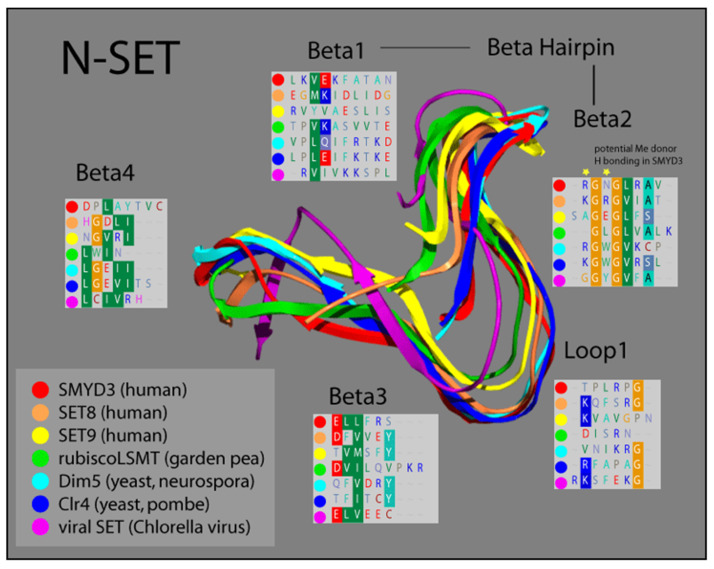
**Ribbon model of N-SET regions of various SET Domain-containing enzymes.** Colors correspond to individual ribbons from different sources sharing the SET-domain protein motifs. Residues in the panels (Beta1-4 and Loop1) correspond to the specific residues in each structural element, highlighting the homology in this region [16,17].

Based on comparisons in sequence conservation, and buttressed by our analysis of its crystal structure, we demonstrated that the SET domain of SMYD3 consists of two non-contiguous elements derived from the N- and C-terminal halves of the SET sequence. Termed N-SET and C-SET, respectively, these are highly conserved, and display remarkable structural overlap with other known SET domains. We overlayed our solved SMYD3 N-SET domain with those of other SET proteins, and found that N-SET domains in humans, plants, yeast, and viruses are composed of four β-sheets linked by a hairpin, between β-1 and β-2, and a loop, between β-2 and β-3 (Figure 2).

**Figure 3:**
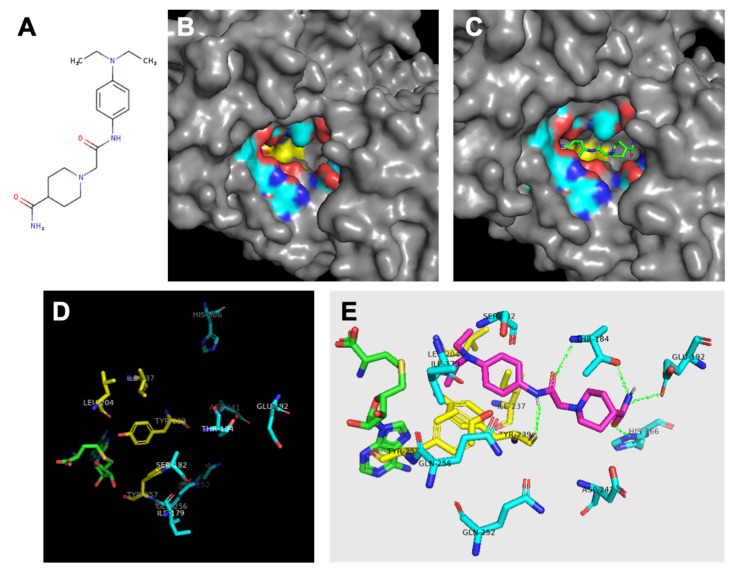
**Analysis of SMYD3 binding pocket and predicted inhibitor interactions.** Light blue indicates carbons in hydrophilic amino acids, yellow indiiates carbons in hydrophobic amino acids. (**A**) Structure of Inhibitor-4. (**B**) Surface model indicating oily hydrophobic core of binding pocket beneath hydrophilic residues. (**C**) Predicted orientation of Inhibitor-4 in binding pocket. (**D**) Significant residues in binding pocket reveal hydrophobic region (yellow) at interface with cofactor SAH (green) followed by a ring of hydrophilic residues. (**E**) Inhibitor-4 (magenta) predicted interactions with binding pocket residues. Predicted hydrogen bonds shown in green.

The catalytic function of SET proteins is dependent upon their ability to transiently interact with an AdoMet cofactor. The crystal structure of other SET proteins has been solved in the presence of this cofactor, and a cluster of residues in the N-SET has been shown to cooperate with another conserved cluster in the C-SET to facilitate this AdoMet interaction [24,30]. Residues 14–19 in this region of SMYD3 align both sequentially and structurally with the N-SET cofactor binding cluster of other SET proteins. Accordingly, our crystal structure analysis of SMYD3 indicates that residues R14 and N16 of SMYD3 (β-2) are positioned such that they would likely contact the AdoMet cofactor, were it positioned as it is within the structure of SET proteins with which AdoMet has been co-crystallized (Figure 2, Beta2 insert, yellow stars). Interestingly, a catalytically critical tyrosine residue that is normally observed in the region corresponding with β-3 of the N-SET in SET domain-containing proteins has been the subject of a unique shift: 150 amino acids toward the C-terminus, in SMYD3. Manifest as F183 in SMYD3, the impact of this residue on the SMYD3 structure corresponds with that of the highly conserved tyrosine present in the N-SET of most other SET proteins (Figure 3).

In addition to the catalytic residue, F183, transposed from the N-SET, the C-SET region consists of two clusters that are absolutely essential for the catalytic activity of SET-dependent methyltransferases [14,31]. These sequences encompass a loop that is required for cofactor binding. The tyrosine of the downstream cluster (Y239 in SMYD3, Figure 3) is invariant among SET-containing methyltransferases [30], and is properly positioned to serve as a base to facilitate the deprotonation of the lysine substrate prior to its methylation.

Finally, the post-SET region of SET-dependent methyltransferases functions as a critical component of the active site by supplying an aromatic residue (Y257 in SMYD3) that anchors against the conserved SET core to form a hydrophobic channel. This channel is a common feature among SET-containing proteins, and serves as the interface along which the substrate associates [25]. Proximal to Y257, SMYD3 contains three cysteines that coordinate with a zinc ion near the catalytic site (Figure 3). Similar to SMYD3, the post-SET of another histone methyltransferase, Dim-5, manifests three zinc-coordinating cysteines that are required for its catalytic activity.

### 3.2. SMYD3 I-SET Domain Resembles Long I-SET of Rubisco-LSMT

The structural similarity of the N-SET and C-SET regions is contrasted by a highly variable insert region (I-SET) in SET proteins. Positioned between the N-SET and the C-SET, the I-SET is a common feature among SET-domain proteins [30]. Although it exhibits considerable variation among SET family members in regard to both structure and length, the I-SET is considered essential for SET-dependent enzymatic activity. This is supported by both biochemical deletion studies [19], as well as structural analyses in which I-SET residues of SET proteins have been shown to be catalytically committed at the substrate interface [24]. In addition to its direct involvement with substrate interactions, the I-SET is thought to stabilize the folded architecture required for the association between the non-contiguous N-SET and C-SET regions [30].

**Figure 4:**
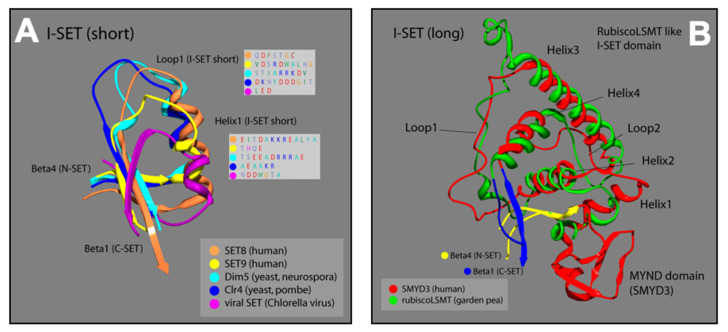
Ribbon models of I-SET regions from multiple SET-domain proteins; colors correspond to labels in key for each ribbon strand. (**A**) Loop1 and Helix1 boxes are predicted protein sequences corresponding to the location of Loop1 and Helix1 of I-SET [16,17]. (**B**) Plant and animal comparison with corresponding colors. Overlay of SMYD3 and garden pea I-SET domains. Distinctive homoloy illustrated in the helix and loop patterns [16,17].

The I-SET structure of SMYD3 is significantly longer than most I-SET domains (Figure 4A). In other human SET proteins (SET8, SET9), as well as in yeast (Dim5, Clr4), and viruses (viral SET), the N-terminal end of the common I-SET originates at the termination of N-SET, β-4, with a short helix followed by a loop that runs into β-1 of the C-SET. The I-SET subdomains of SMYD proteins, however, are preceded by the MYND domain, which is the primary element of distinction for the SMYD proteins among other SET families. From the C-terminal MYND, the SMYD3 I-SET initiates with a short helix, and proceeds through a series of three additional helices and two loops, before terminating at β-1 of the C-SET. Fascinatingly, the SMYD3 I-SET domain contains four helices connected by two loops, and is nearly identical to the I-SET domain in the ancient Rubisco-large subunit methyltransferase (Rubisco-LSMT) (Figure 4B). As its name implies, Rubisco-LSMT methylates K14 on the large subunit of the Rubisco holoenzyme, which is involved in the rate-limiting CO_2_ fixation step of photosynthesis [23,32]. The Rubisco enzyme is the most prevalent enzyme in plants and, by extension, on Earth. The uniqueness of the SMYD3 I-SET (Rubisco-LSMT-like) domain, and its similarity to the ancient Rubisco-LSMT enzyme, speaks to the fine-tuned design of the subdomain, and makes it a promising region of SMYD3 to target for specific inhibition within the SMYD family.

Though the I-SET region of SMYD3 does not appear to interact with the substrate near its methylation site, we predict that it may contact the substrate further upstream, as the I-SET comprises part of a groove along which the substrate extends (Figure 1C). Additionally, our analysis of the I-SET interaction potential, derived from charge and proximity indices, demonstrates that it is likely involved with cofactor binding at the active site of SMYD3 (Figure 1B).

### 3.3. SMYD3 MYND Domain Highly Similar to Other MYND Enzymes

Similar to the N-SET and C-SET regions, the zinc finger-containing MYND domain in SMYD3 closely resembles MYND domains in other human proteins. The motif contains two zZinc-binding sites at either end of an alpha-helix that are stabilized by the presence of seven invariant cysteine residues, and an invariant histidine residue (Figure 4). A point mutation in any of these critical residues disrupts the MYND structure, and abolishes MYND-mediated protein interactions [33]. We compared the crystal structure of the SMYD3 MYND domain with those of ZMYND10 and ETO, and concluded that the basic architecture of these MYND domains is almost indistinguishable (Figure 3). The structural similarities of the N-SET and MYND domains among these proteins are fascinating and speak to the high utility of the structure in protein-–protein interactions, overall enzyme stability, and definition of the cofactor binding pocket. However, because of the lack of specificity to SMYD3, neither domain represents a promising region of the enzyme for small molecule inhibitor targeting.

### 3.4. Analysis of Binding Pocket Reveals Hydrophobic Core and Hydrophilic Ring

**Figure 5:**
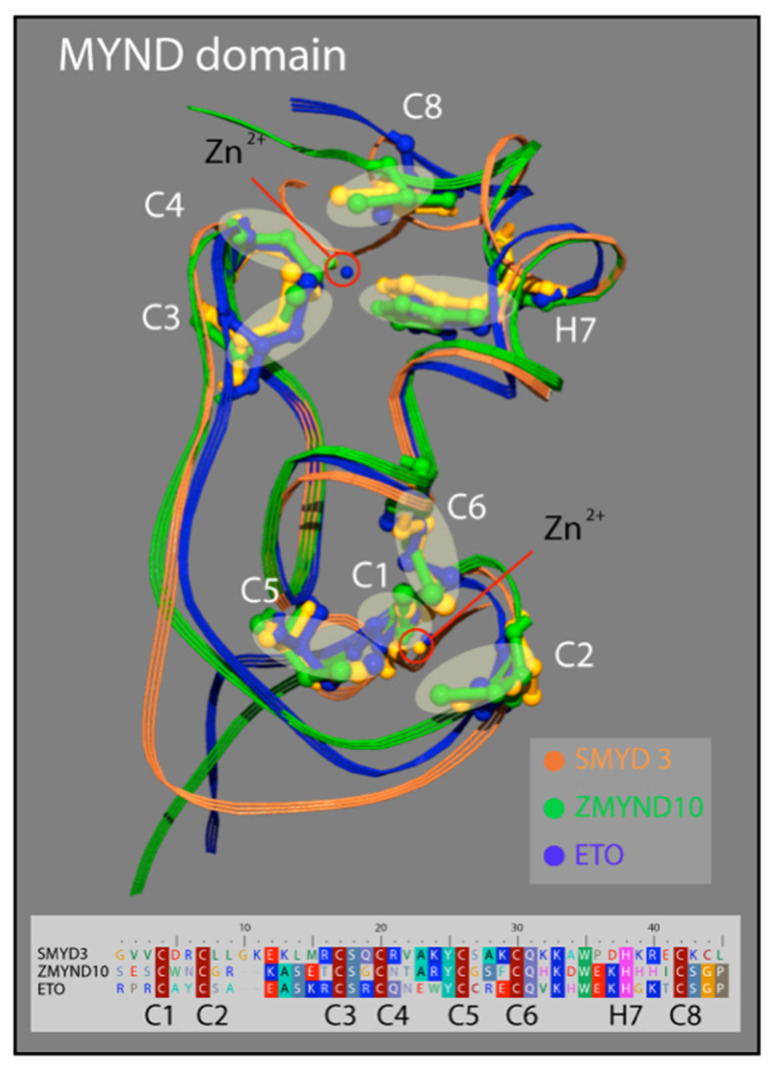
Structure of the MYND domain. A ribbon representation of the secondary structure for the MYND domain of SMYD3 (orange) is superimposed on the MYND domains of ZMYND10 (green) and ETO (blue). An insert shows a sequence alignment for the MYND domains of these three proteins. The seven invariant cysteine residues (red) and the invariant histidine (pink) are included.

In total, the protein ligand binding pocket is composed of parts of the C-SET, post-SET, and I-SET subdomains. Specific analysis of the hydrophobicity and hydrophilicity of the binding pocket was conducted to inform inhibitor design, and to aid in predicting the inhibitor binding location within the pocket. If the enzyme binding pocket is thought of as a conical funnel (the opening at the bottom being the catalytic interface between the protein substrate and SAM/SAH), it was found that the bottom-most ring of the funnel consists of almost entirely hydrophobic residues F183, I237, L204, I214, Y239, and Y257. The next-highest concentric ring of the funnel consists of almost entirely hydrophilic residues Q256, Q252, S182, T184, Q192, H366, P241, and the amino/carboxyl backbone groups of I179 and Y239. This arrangement yields a polar region around the middle of the binding pocket, and an oily region at the bottom of the binding pocket that could be exploited for inhibitor design (Figure 3B,D). As previously described [19], SMYD3 small molecule inhibitors were designed in silico to competitively inhibit the protein substrate binding pocket of SMYD3. We previously demonstrated that one of these inhibitors, Inhibitor-4 (Figure 5A), blocks SMYD3 catalytic activity in a methyltransferase assay [19]. Here, we proceeded to analyze the predicted binding location of Inhibitor-4 within SMYD3 in order to inform future design iterations using structure–activity analysis. Inhibitor-4 appears to sit deep within the binding pocket (Figure 5C), interacting primarily with the C-SET subdomain. The terminal pair of hydrophobic ethyl groups, and the benzene ring on Inhibitor-4 give the compound amphipathic properties, allowing it to favorably straddle the hydrophobic core of the pocket, and the hydrophilic ring above (Figure 5E). On the polar end, Inhibitor-4 likely forms hydrogen bonds with T184, Q192, H366, and Y239.

### 3.5. In Silico-Designed SMYD3 Inhibitor Reduces Viability of Lung Cancer and Colorectal Cancer Cell Lines

**Figure 6:**
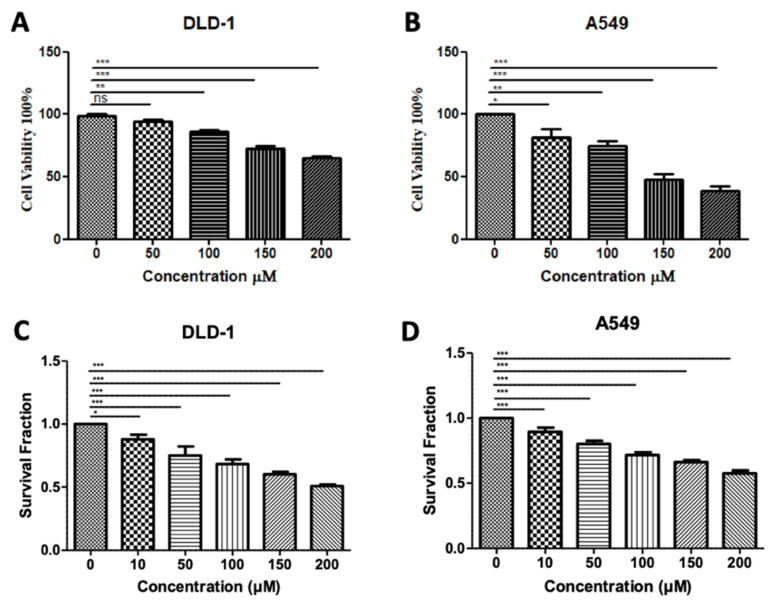
**Treatment with SMYD3 Inhibitor-4 reduces carcinoma cell viability and proliferation.** Cell viability of DLD-1 colorectal cancer cells (**A**) and A549 lung cancer cells (**B**) after 48 h of treatment with various concentrations. Survival fraction/proliferation of DLD-1 cells (**C**) and A549 lung cancer cells (**D**) after two weeks of treatment with various concetnratons of Inhibitor-4.

In our previous testing of Inhibitor-4, we demonstrated its ability to reduce the proliferation and viability of SMYD3-positive breast cancer cells without impacting wild-type breast epithelial cells [19]. Using similar methodology, we demonstrated here that treatment of colorectal and lung carcinoma cell lines with Inhibitor-4 significantly reduces the cell viability (Figure 6A,B) and proliferation (Figure 6C,D) in a dose-responsive manner. The computational analysis shown in this work supports the experimental in vitro studies, which identified Inhibitor-4 to significantly inhibit SMYD3 methyltransferase activity.

## 4. Discussion

Epigenetic control is tightly regulated in humans, and aberrant epigenetic marks are associated with several disease pathologies. Histone lysine methylation is a key epigenetic regulator, and is modulated chiefly by SET-domain-containing enzymes. Specifically, SMYD3 is a SET- and MYND-domain-containing enzyme that methylates both histone and non-histone targets. The overexpression of SMYD3 has been discovered in several cancer types, including breast, colorectal, and lung carcinomas. The inhibition of SMYD3 is promising for the therapeutic treatment of these cancer types.

Structural overlays of similar proteins and protein domains, such as the overlays presented here, allow for in-depth analysis of functional residues, secondary structures, and enzyme binding pockets. This analysis sheds light on the similarities and differences in catalytic mechanisms across species, and can also inform the development of inhibitors for therapeutic intervention, or the study of protein function. Notably, our analysis revealed that the I-SET domain of SMYD3 is unique among other SET proteins, closely resembling the long I-SET domain of the ancient plant methyltransferase, Rubisco-LMNT.

We previously developed a small molecule SMYD3 inhibitor using a random screen in silico, and demonstrated its efficacy in limiting the growth and survival of breast cancer cells (Inibitor-4, [19]). In this work, we examined the structural features of SMYD3 that interact favorably with Inhibitor-4, and also assessed its effects on colorectal and lung cancer cell lines, which, along with breast cancer lines, have been shown to overexpress SMYD3. Future design could seek to increase the amphipathicity of the molecule to better capitalize on the polar structure of the binding pocket. Additionally, because of SMYD3′s specific resemblance to Rubisco-LMNT, known Rubisco inhibitors could be tested as potential SMYD3 inhibitors.

The DLD-1 colorectal cancer cell line has been shown to express lower levels of SMYD3 than some other colorectal lines [18], which may explain the high dose of Inhibitor-4 (200 µM) needed to reduce the cell viability and proliferation levels by 30 and 50 percent, respectively. However, even at 100 µM, significant decreases were observed in both cell viability and proliferation. No decrease was observed in DLD-1 cells when treated with 100 µM BCI-121, a previously-developed inhibitor for SMYD3 [18]. BCI-121 and Inhibitor-4 contain structural similarity; however, the benzene ring of BCI-121 has a bromine substituent, whereas the benzene ring in Inhibitor-4 has a diethyl amine substituent. The two ethyl groups increase the hydrophobicity of Inhibitor-4, and are predicted to interact favorably with L290 and Y326, two hydrophobic residues that contribute to alpha-helices in the C-SET domain.

## 5. Conclusions

We conclude that Inhibitor-4 is a promising inhibitor of SMYD3 and SMYD3-mediated breast, lung, and colorectal cancers. The structure–activity relationships explored here will inform compound modifications, and optimization of the inhibitor. Future work will also include the development of inhibitors for other enzymes implicated in disease, with an emphasis on enzymes that modulate the epigenetic landscapes of cells.

## Data Availability

Data available upon request to authors.
